# Patterns of financial incentives in primary healthcare settings in Nigeria: implications for the productivity of frontline health workers

**DOI:** 10.1186/s13104-021-05671-z

**Published:** 2021-06-30

**Authors:** Ekechi Okereke, George Eluwa, Akinwumi Akinola, Ibrahim Suleiman, Godwin Unumeri, Sylvia Adebajo

**Affiliations:** Population Council, 16 Mafemi Crescent, off Solomon Lar Way, Utako, Abuja, Nigeria

**Keywords:** Incentives, Frontline health workers, Productivity, Primary healthcare, Nigeria

## Abstract

**Objective:**

This study was designed to explore the patterns of financial incentives received by some frontline health workers (including nurses, midwives as well as community health workers in paid employment) and the implications for their productivity within rural settings in Nigeria. A cross-sectional quantitative design in two States in Nigeria was adopted. Structured interviews were conducted with 114 frontline health workers. Bivariate analysis and multivariate regression analysis were carried out to explore relationships between the satisfaction of frontline health workers with the financial incentives received and their productivity in rural settings as well as the extent of any such relationships.

**Results:**

Bivariate analysis demonstrated a statistically significant relationship (P = 0.013) between satisfaction with incentives received by frontline health workers and their productivity in rural settings. When other predictors were controlled for within a multivariate regression model, those who received incentives and were satisfied with the incentives were about three times more likely to be more productive at work than those who were unsatisfied with incentives (AOR: 3.3; P = 0.009, 95% CI = 1.3–8.2). In conclusion, the determination of type and content of incentives should be done in consultation with *all* relevant stakeholders, including possibly a cross-section of health workers themselves.

**Supplementary Information:**

The online version contains supplementary material available at 10.1186/s13104-021-05671-z.

## Introduction

A properly motivated health workforce is a prerequisite for effective maternal, newborn and child health (MNCH) service delivery [1, 2, 3]. Globally, there are challenges associated with improving the productivity and retention of frontline health workers especially in rural communities [4]. The factors that influence the productivity of frontline health workers within primary healthcare settings in low- and middle-income countries need to be further researched and documented.

Nigeria, the most populous country in sub-Saharan Africa has poor maternal, newborn and child morbidity and mortality indices [5, 6]. The country’s infant and under-five mortality rates from the 2018 National Demographic and Health Survey (NDHS) indicate that infant and under-5 mortality rates are 67 and 132 deaths per 1000 live births respectively [7]. The Nigerian health system is divided into the tertiary, secondary and primary healthcare systems, depending on which level of government is responsible for management, the type of healthcare services provided as well as healthcare personnel available to provide healthcare services. The tertiary healthcare system is managed and resourced by the Federal Government, the secondary health system is managed and resourced by the respective State governments, while the primary health system is managed by the Local governments. Although nurses and midwives are available at all levels of the healthcare system in Nigeria, community health workers i.e., community health officers (CHOs), community health extension workers (CHEWs) and Junior community health workers (JCHEWs) are only available and provide healthcare services at the primary healthcare level [8].

According to National Guidelines for the development of primary healthcare systems in Nigeria, JCHEWs are expected to provide essential community-based services such as promoting the community’s participation in health-related activities, conducting home visits and clients’ follow up as well as identifying and registering pregnant women for antenatal care. The JCHEWs should spend about 90% of their time working within the community but in addition they provide some basic healthcare services within assigned health facilities, especially in rural communities. The CHEWs have similar community-based functions as the JCHEWs but tend to spend less time (about 40% or less of their time) working in the community and more time working within health facilities, promoting maternal and child healthcare and managing patients according to basic clinical protocols. The community health officer tends to have more administrative roles within the health facility, but with similar community-based functions and facility-based maternal newborn and child healthcare (MNCH) responsibilities as the CHEWs [9].

The primary health care system in Nigeria is inadequately financed and poorly functioning with dire consequences for frontline health workers’ productivity at primary healthcare level [10]. One of the key strategies for increasing frontline health workforce availability and productivity within rural communities is through the introduction and use of incentives [11, 12, 13]. This is more so, when remunerations for health workers are poor and insufficient to cover the most basic needs of health workers and their families [14, 15]. Increasing salaries is not a ‘magic bullet’ for dealing with the problem of poor availability and productivity of frontline health workers, because better salaries do not necessarily translate to increased productivity and retention among health workers [3].

The human resources for health (HRH) project in Nigeria was implemented by Population Council and the World Health Organization (WHO). The HRH project carried out a study in some rural communities within Bauchi and Cross River states to assess the implications of financial incentives on the productivity of specific frontline health workers as well as to investigate the relationship between satisfaction with the financial incentives received by these frontline health workers and their productivity in rural primary healthcare settings in Nigeria. Frontline health workers as defined within the HRH project and applied to this research study refer to nurses, midwives and community health workers i.e. CHEWs, JCHEWs and CHOs.

## Main text

### Methods

#### Study sites

This study was conducted in *Alkaleri* and *Giade* local government areas (LGAs) in Bauchi State in North-East Nigeria as well as *Etung* and *Yala* LGAs in Cross River State, within the southern part of the country. The study was undertaken in Bauchi and Cross-river states because these were the HRH project focal states in Nigeria.

#### Study design and sampling procedure

The study employed a cross-sectional quantitative research design. To conduct the sampling for the study, the list of LGAs in Bauchi and Cross River states were stratified into urban and rural. This was followed by a random selection of two rural LGAs per State—Alkaleri and Giade LGAs in Bauchi state; Yala and Etung LGAs in Cross River state using the compiled list of rural LGAs for each state as the sampling frame. A list of PHC facilities which offered maternal, newborn and child health services was stratified as Health posts, Primary Health Clinics and Primary Healthcare Centres from which an equal representation of these different types of facilities were selected.

One hundred and fourteen (114) frontline health workers i.e. community health extension workers (CHEW), junior community health extension workers (JCHEW), community health officers (CHOs), nurses and midwives were selected from sixty-six (66) randomly selected primary healthcare facilities in Bauchi and Cross River states. These 66 health facilities represent half of all available primary healthcare facilities in the randomly selected rural LGAs within the study states, based on a sampling approach used by Adeniyi and colleagues [16].

#### Data collection and management

Data collection was undertaken by monitoring and evaluation (M&E) officers from selected Local Government Areas (LGAs) in each state, who were trained on the use of the study questionnaire (see sample as Additional file 2) as well as on research ethics. The study questionnaires were first pilot-tested, then the pre-tested questionnaires were uploaded on personal digital assistants (PDAs) and used to collect self-reported data from the survey respondents. Data analysis was done using SPSS software.

#### Definition and measurement of productivity of FLHWs for this study

Financial incentives for this study were defined as additional monetary payments that contribute to health workforce productivity. The World Health Organization has identified productivity as one of the outcomes to measure health workforce performance [17]. Jaskiewicz W and her colleague defined productivity as healthcare services provided by a healthcare worker over a given period of time [18]. However, productivity for this study is defined as the number of patients/clients to which a frontline health worker provides healthcare services on a daily basis for a full time equivalent (FTE) of 40 h/week. Consequently, productivity for this study was measured using two proxies: (1) average number of patients/clients seen daily by a frontline health worker (client load) and (2) number of hours of work per week by a frontline health worker (level of effort). Calculations using data collected from the health facilities involved in the study estimated ‘7 patients per day’ as the average number of patients attended by frontline health workers per day. Thus, a frontline health worker who attended to over 7 patients per day was categorized as having ‘adequate’ client load while those who attended to 7 patients and below on average per day were categorized as having inadequate client load. Furthermore, the minimum weekly hours of work/level of effort required for frontline health workers was estimated at 40 h/week, hence frontline health workers who spent 40 h or more at their work were categorized as having ‘adequate’ level of effort while those who spent below 40 h/week were categorized as having ‘inadequate’ level of effort. Frontline health workers with adequate client load and simultaneously having adequate level of effort were categorized as frontline health workers with adequate productivity, others were classified as frontline health workers with inadequate productivity.

#### Ethical considerations

Ethical approval was granted by Bauchi and Cross River States’ Research Ethical Committees and Population Council’s Institutional Review Board (IRB). Written informed consent was sought and obtained from each study respondent prior to starting the interviews for the study.

### Results

#### Key characteristics of frontline health workers

A total of 114 frontline health workers from Bauchi (43%) and Cross River state (57%) were enrolled into this study. The majority (over 85%) were 30 years and above and married (91%) while more than half (59%) were females. CHEWs constituted the largest (47%) proportion of respondents, followed by JCHEWs (45%) while nurses (1%) and midwives (2%) were few working at primary healthcare level in both states.

#### Incentives received by frontline health workers at primary health care facilities

Over half (51.8%) of the frontline health workers received some form of financial incentive, but majority (61.4%) were unsatisfied with the incentives which they received (Table 1). The types of financial incentives received ranged from rural posting allowance (66%); stipends for adhoc jobs such as immunization (20%); loans (20%); per diem for conference attendance (35%) or trainings (40%). About a quarter (27%) and 3% reported receiving reimbursements for transport fare and money for referrals respectively as financial incentives. Beyond their primary occupation as health care providers, most frontline health workers engaged in farming (70%) or petty trading (16%). A few operated as patent medicine vendors (6%) and home birth attendants (9%) to augment their incomes (see Additional file 1: Tables S1 and S2).

**Table 1:** Key characteristics of frontline health workers enrolled into the study (N = 114)

Characteristic	N	%
State		
Bauchi	49	43.0
Cross River	65	57.0
Sex		
Male	47	41.2
Female	67	58.8
Age		
≤ 29 yrs	15	13.2
30–39 yrs	51	44.7
40 yrs above	48	42.1
Marital status		
Married	104	91.2
Single/widowed	10	8.8
Religion		
Christianity	76	66.7
Islam	38	33.3
Education		
Post-secondary school	112	98.2
University degree and above	2	1.8
Type of FLHW		
JCHEW	51	44.7
CHEW	53	46.5
CHO	7	6.1
Nurse	1	0.9
Midwife	2	1.8
Years in PHC		
< 2 years	30	26.3
2–4 years	45	39.5
5 yrs and above	39	34.2
Years in current position		
< 2 yrs	34	29.8
2–4 yrs	49	43.0
5 yrs and above	31	27.2
Received financial incentive		
Yes	59	51.8
No	55	48.2
Satisfaction with incentives		
Satisfied	44	38.6
Unsatisfied	70	61.4

#### Bivariate analysis

Bivariate analysis was carried out to explore relationships between frontline health workers’ satisfaction with incentives and their productivity within rural primary healthcare settings. There was a statistically significant relationship (P = 0.013) between frontline health workers’ satisfaction with incentives and their productivity in rural primary healthcare settings. Adequate productivity was higher (46% vs. 23%) among frontline health workers who reported being satisfied with the incentives compared to those who reported not being satisfied with incentives (Table 2).

**Table 2:** Bivariate analysis between satisfaction with incentives received for MNCH work and health workforce productivity at PHC level

Characteristic	Inadequate productivity (%)	Adequate productivity (%)	X^2^	P value
Level of satisfaction with incentives for MNCH work				
Satisfied	54.5	45.5	6.385	0.013
Unsatisfied	77.1	22.9		

#### Multivariate analysis

To ascertain the extent of the relationship between satisfaction with incentives and frontline health workforce productivity, binomial regression was conducted as shown in Table 3. Results from unadjusted regression reveals that health workers who were satisfied with incentives were 2.8 times more likely to demonstrate better productivity at their work than those who were unsatisfied with the incentives (P = 0.013, CI = 1.3–6.3). When other predictors were controlled for, those who were satisfied with incentives were 3.3 times more likely to be more productive at their work than those who are unsatisfied with incentives (P = 0.009, CI = 1.3–8.2).

**Table 3:** Predictors of frontline health workers’ productivity at primary healthcare level

Characteristic	Univariate analysis OR (95% CI)	Multivariate analysis OR (95% CI)
Facility type	0.193	0181
Health clinic	Ref.	Ref.
Health post	0.4 (0.1–1.5)	0.5 (0.1–2.0)
PHC	1.1 (0.4–3.1)	1.5 (0.5–4.6)
Sex	0.091	0.067
Female	Ref.	Ref.
Male	1.9 (0.9–4.5)	2.3 (0.9–5.7)
Age	0.735	0.957
> 4 yrs	Ref.	Ref.
30–3 yrs	1.1 (0.5–2.6)	1.2 (0.3–4.5)
< 2 yrs	1.6 (0.5–5.4)	1.0 (0.4–2.7)
Education	0.581	0.959
Post-sec school	Ref.	Ref.
University degree and above	2.2 (0.1–36.1)	0.9 (0.1–18.3)
Satisfaction with incentive for MNCH work	< 0.013	0.009
Unsatisfactory incentive	Ref.	Ref.
Satisfactory incentive	2.8 (1.3–6.3)	3.3 (1.3–8.23)

### Discussion

This study was designed to explore the financial incentives received by some frontline health workers (nurses, midwives and community health workers) which could influence their availability and productivity with possible consequences for primary healthcare service delivery in Nigeria. Community health workers constitute over 95% of the sample of frontline health workers who were involved in this study. Current financial incentives within primary healthcare settings in Nigeria may be more applicable to these cadres of frontline health workers. Nurses and midwives were scarce within the primary healthcare facilities involved in this study and this is largely reflective of the current dearth of nurses and midwives in most health facilities within rural communities and in primary healthcare settings across Nigeria. This finding raises concerns about the geographical imbalances of frontline health workers and its impact on delivering universal healthcare [19]. It is likely that the majority of the current financial incentives available at this level of care—primary healthcare, may not be sufficient enough to adequately retain nurses and midwives. In addition, nurses and midwives appear to have better opportunities to migrate to higher levels of the healthcare delivery system, where more lucrative financial and non-financial incentive mechanisms exist.

Slightly over half (52%) of the frontline health workers enrolled in this study received some form of financial incentive while working in rural and primary healthcare settings. Around two-thirds of frontline health workers involved in this study received rural posting allowance. However, it is imperative to give rural posting allowance to every frontline health worker working in rural communities to incentivize them to take up the responsibility to provide healthcare services and remain working within rural and primary healthcare settings [4]. Rural posting allowance for frontline health workers should be attractive enough to encourage/persuade health workers to ignore the opportunities which are available in urban settings and encourage their retention in rural areas. State and Federal governments should possibly introduce policies which encourage private sector organizations to apply their corporate social responsibilities towards initiatives which motivate frontline health workers for example by introducing different types of incentives. Various stakeholders including donors such as the World Bank, should collaborate with governments at different levels to introduce, implement and sustain incentives [10] to improve the productivity of frontline health workers in rural and primary healthcare settings.

Possible consequences when the salaries and income of frontline health workers are not enough could include scenarios where health workers seek alternative sources of income, sometimes to the detriment of healthcare services provided to patients and clients [1]. The study findings indicate that about 70% of respondents engage in farming as an alternative source of income—this is not unusual considering that majority of these frontline health workers reside in rural communities with a lot of agricultural land. About 16% of the study respondents indicated involvement in some form of petty trading, with some health workers providing patent medicine (6%) and home birth attendance services (9%). These findings which illustrate that frontline health workers get involved in other economic activities to supplement their income is consistent with what has been reported elsewhere—Akwataghibe and colleagues reported that out of 165 respondents, over half of health workers indicated having additional medical or non-medical earning arrangements, the majority (over 40%) reported involvement in non-medical activities such as farming and trading [14].

Bivariate analysis suggests that satisfaction of frontline health workers with financial incentives has a relationship with their productivity. In other words, if frontline health workers perceive/regard the incentives which they receive as satisfactory, then this will very likely improve their productivity. Multivariate regression analysis indicated that those who received incentives and were satisfied with the incentives were about three times likely to be more productive at their work than those who were unsatisfied with incentives. These findings strengthen the argument for the need to introduce, implement and sustain context-appropriate incentives which frontline health workers regard as satisfactory to encourage their retention in rural communities as well as improve their productivity. However, to develop interventions which promote a high productivity workforce, requires a proper understanding of the current HRH situation and effective management and planning practices [20].

It is important that there are necessary consultations with all the relevant stakeholders about the type and content of incentives [21], including possibly with a cross-section of health workers or the professional associations of health workers who receive the incentives. A comprehensive strategy for improving frontline health workforce productivity should ideally include a mixture of financial and non-financial incentives [10] as well as consider the human resources for health situation within rural and primary healthcare settings, particularly for low- and middle-income countries [21].

## Limitations

The study has some limitations such as the possibility of response bias among study respondents. In addition, the study has a relatively small sample size from across the different cadres of frontline health workers at primary healthcare level—nurses, midwives, community health officers, community health extension workers and junior community health extension workers.

## Supplementary Information

**Additional file 1: Table S1.** Major sources and types of financial incentives for frontline health workers (N = 59). **Table S2.** Other economic activities of frontline health workers (N = 114).

**Additional file 2.** Frontline Health Worker Questionnaire.

## Data Availability

The datasets for this study are available from the corresponding author upon reasonable request.

## Abbreviations

CHEW: Community health extension worker
CHO: Community health officer
FLHW: Frontline health worker
GAC: Global Affairs Canada
HRH: Human resources for health
IRB: Institutional review board
JCHEW: Junior community health extension worker
LGA: Local government area
MNCH: Maternal newborn and child health
NDHS: National demographic and health survey
PDA: Personal digital assistant
PHC: Primary health care
SPSS: Statistical package for social sciences
WHO: World Health Organization
