# Supplementary material for: Patterns of financial incentives in primary healthcare settings in Nigeria: implications for the productivity of frontline health workers
Source: BMC Res Notes. 2021 Jun 30;14:250. doi: 10.1186/s13104-021-05671-z (PMC8243849; doi:10.1186/s13104-021-05671-z)
Supplement: Supplementary file 1 — Additional file 1: Table S1. Major sources and types of financial incentives for frontline health workers (N = 59). Table S2. Other economic activities of frontline health workers (N = 114). [file 13104_2021_5671_MOESM1_ESM.docx]

**Table S1: Major sources and types of financial incentives for frontline health workers (N = 59)**

| **Characteristic** | **N** | **%** |
| --- | --- | --- |
| **Major sources of financial incentives**  Local Government | 25 | 42.4 |
| State Government | 7 | 11.9 |
| Community/Village | 16 | 27.1 |
| Private Organization/NGO  **Types of financial incentives received by FLHWs**  Rural posting allowance  Stipend for adhoc job e.g. immunization  Loan  Per diem for conference attendance  Reimbursement for transport fare  Per diem for training attendance  Money for referrals | 11  39  13  11  21  16  23  2 | 18.6  66.1  22.0  18.6  35.6    27.1  39.0  3.4 |

**Table S2: Other economic activities of frontline health workers (N=114)**

| **Characteristic** | **N** | **%** |
| --- | --- | --- |
|  |  |  |
| Farming | 79 | 69.3 |
| Petty Trading | 18 | 15.8 |
| Chemist/Patent Medicine Vendor | 7 | 6.1 |
| Home birth attendance service | 10 | 8.8 |
|  |  |  |
