# Supplementary material for: Patterns of financial incentives in primary healthcare settings in Nigeria: implications for the productivity of frontline health workers
Source: BMC Res Notes. 2021 Jun 30;14:250. doi: 10.1186/s13104-021-05671-z (PMC8243849; doi:10.1186/s13104-021-05671-z)
Supplement: Supplementary file 2 — Additional file 2. Frontline Health Worker Questionnaire. [file 13104_2021_5671_MOESM2_ESM.docx]

**Frontline Health Worker Questionnaire Date _______________________________**

**General guidelines to interviewers:**

1. Identify frontline health workers who are either nurses, midwives or community health workers in the health facility.
2. Introduce yourself and ask if you can ask a few questions which will not take more than 30 minutes. Explain that the answers to the questions will help to improve the conditions of service for frontline health workers who provide health services in rural settings.
3. If a frontline health worker agrees to be interviewed, start the interview with the following preamble:

*You are being invited to take part in a research study. This study will involve interviewing some cadres of frontline health workers (nurses, midwives and community health workers), who work in primary healthcare facilities in rural settings. At the end of the study, there will be recommendations for the government, donors and policy-makers on how to better improve the conditions of service for these frontline health workers. This is part of a larger study which aims to better understand primary healthcare service delivery. The interview for this study will last for not more than 30 minutes.*

*Before you decide whether to participate, you may need to understand why the research is being done and what it would involve. Please ask me if there is anything that is not clear. If you feel that you understand the purpose of this study or when all of your questions have been answered (if you have any questions), you will be asked if you wish to participate in the study, and if yes, to sign an ‘Informed Consent Form’. You will be given a signed copy to keep.*

Do you agree to participate in this interview for the study?

Yes ___________

No ___________

***Instructions:***

1. ***Consent Instructions for Interviewer: Give a copy of the ‘Informed Consent Form’ to the frontline health worker or read the standard consent text from the Informed Consent Form if the frontline health worker agrees to participate in the study. Each frontline health worker who agrees to participate must sign the informed consent form.***
2. ***Interviewer should complete the frontline health worker questionnaire.***

Name of Primary Healthcare Facility Location of Primary Healthcare Facility (LGA/State)

__________________________________ ________________________/________________

**Section 1: Socio-Demographic Information**

1. **Sex of respondent**

_ Male

_ Female

1. **What is your current age in years?**

___________________________

1. **What is your marital status?**

_ Married

_ Single

_ Divorced

_ Widowed

_ Seperated

1. **Religion**

_ Christianity

_ Islam

_ Traditional religion

1. **Highest Academic Qualification**

*Post-secondary school qualification*

_ Certificate

_ Diploma

_ Higher Diploma

_ Nurse

_ Midwife

_ Nurse and Midwife

*University qualification*

_ University level (B.Sc.)

_ Postgraduate

1. **Type of Frontline Health Worker (FLHW)**

_ Junior CHEW (JCHEW)

_ Senior CHEW (CHEW)

_ Community Health Officer (CHO)

_ Nurse

_ Midwife

1. **How long have you worked at the Primary Health Care level?**

**___________________________________________**

1. **How long have you worked in your current position?**

**___________________________________________**

**Section 2: Working Conditions**

1. **In your work as a nurse, midwife, CHO, CHEW or JCHEW, how many patients, on average, are you responsible for in a day?**

**___________________________________________**

1. **Do you feel that this is too few, too many, or just the right amount?**

_ Too Few

_ Too many

_ Just right

1. **How many households within the community did you visit in the past month?**

**___________________________________________**

1. **Do you feel that this is too few, too many, or just the right amount?**

_ Too Few

_ Too many

_ Just right

1. **How much time (in hours) do you spend each week on your work?**

**____________________________________________**

1. **Do you feel that this is too few, too many, or just the right amount?**

_ Too Few

_ Too many

_ Just right

1. **What mode of transport do you most often use to travel to the health facility?**

_ Walking _ Bicycle

_ Motor cycle _ Motor Vehicle (truck/car/tricycle)

**Section 3: Economic Activities**

1. **Do you or did you receive any financial incentives for your maternal newborn and child health (MNCH) work?**

_Yes

_No

1. **What is (was) the primary source of these incentives?**

_ Local Government

_ State Government

_ Federal Government

_ NGO

_ Community/Village

_ Private Organisation

_ Religious Organisation

1. **What type of incentives do you receive?**

READ OPTIONS ALOUD

Yes No

Rural posting allowance ____ ____

Stipend for adhoc jobs e.g. immunization ____ ____

Loan ____ ____

Per diem for conference attendance ____ ____

Per diem for training attendance ____ ____

Reimbursement for transport fare ____ ____

Money for referral ____ ____

1. **Are you satisfied with the financial incentives available and those you have received?**

Yes ____ No____

**Section 4: Other Economic Activities**

1. **What has been your other economic activities beyond your work as frontline health worker in primary healthcare?**

_ Farming

_ Petty Trading

_ Commercial Motorcycling

_ Patent medicine vendor

_ Food Vendor

_ Home Service Birth Attendant
